# Nomogram for Predicting the Overall Survival of Adult Patients With Primary Gastrointestinal Diffuse Large B Cell Lymphoma: A SEER- Based Study

**DOI:** 10.3389/fonc.2020.01093

**Published:** 2020-07-03

**Authors:** Jing Wang, Min Zhou, Rongfu Zhou, Jingyan Xu, Bing Chen

**Affiliations:** Department of Hematology, The Affiliated Drum Tower Hospital of Nanjing University Medical School, Nanjing, China

**Keywords:** gastrointestinal DLBCL, prognosis, nomogram, SEER, survival

## Abstract

**Background:** The aim of this study was to establish a precise prognostic model, based on significant clinical parameters, for predicting the overall survival (OS) of adult patients with primary gastrointestinal diffuse large B cell lymphoma (GI DLBCL).

**Materials and Methods:** The data of 7,121 GI DLBCL patients, diagnosed between 1997 and 2015, were obtained from the Surveillance, Epidemiology, and End Results (SEER) database. These patients were randomly divided into two sequential cohorts: training (*n* = 5,697) set and validation (*n* = 1,424) set. ROC methodology and calibration curves were explicitly used to evaluate the predictive performance of nomogram.

**Results:** The median OS in the training cohort was 76 months (1–239 months), and 3, 5, and 10-year OS rates were 60.3, 53.9, and 39.5%, respectively. Age at diagnosis, Ann Arbor stage, and marital status were important clinical predictors of OS. These characteristics were used to build a nomogram. The AUC of the nomogram for predicting 3, 5, and 10-year OS were 0.669, 0.692, and 0.740, respectively. All RUC and calibration curves revealed good accuracy in predicting prognosis of GI DLBCL.

**Conclusion:** In summary, the established nomogram was validated to predict OS for adult patients with GI DLBCL. This predictive model could help clinicians identify high-risk patients to improve their prognosis.

## Introduction

The primary gastrointestinal (GI) lymphoma is the most common type of extranodal lymphomas, accounting for about 25% of all primary extranodal lymphomas (1). However, primary GI lymphoma constitutes only about 1–4% of all GI cancers (2). More than half the cases occur in the stomach, followed in small intestine and ileocecum (2). Histopathological findings reveal the following types: marginal zone lymphoma (MALT), diffuse large B-cell lymphoma (DLBCL), enteropathy-associated lymphoma (EATL), mantle cell lymphoma (MCL), and others. According to histological type, DLBCL is the most common GI lymphoma with a prevalence estimated at 40–50% (2, 3). The next most common histological type is mucosa-associated lymphoid tissue (MALT) lymphoma (4). Contrary to nodal lymphomas, GI DLBCL has different clinical characteristics and prognosis. C-myc rearrangements which are more common in GI DLBCL than in nodal lymphomas, do not seem to negatively influence the prognosis (6). GI DLBCL is usually diagnosed with low or intermediate International Prognostic Index (IPI). In a retrospective analysis, patients with GI DLBCL showed better overall survival (OS) than patients with nodal or other extranodal sites (7). Nevertheless, only a few small-sample studies are conducted to search for prognostic factors because of the rareness of these tumors in recent years. Recently, Surveillance, Epidemiology, and End Results (SEER) database has been used to identify predictive factors to develop a predictive nomogram to predict the long-term survival. In this study, we use the patient records from the SEER database to establish a novel nomogram to predict the overall survival of adult patients with primary GI DLBCL.

## Methods

### Data Source and Study Population

Data for analysis were extracted from the SEER program of the National Cancer Institute. The SEER program statistical analysis software (SEER^*^Stat, Version 8.3.6) was used to examine the data for adult patients (≥18 years) diagnosed GI DLBCL between 1997 and 2015. In 1997, rituximab became the first targeted drug approved by the FDA for the treatment of B-cell NHL. The era of rituximab has arrived. The following information was obtained for each patient: age at diagnosis, sex, race, marriage, Ann Arbor stage, primary site, surgery, survival time, and status. Patients lacking these characteristics data were excluded from this study. A total of 7,121 adult GI DLBCL patients were randomly divided into two sequential cohorts: training (*n* = 5,697) set and validation (*n* = 1,424) set. Marriage of patients was recorded as married and single (never married, divorced, and widowed).

### Construction and Validation of the Nomogram

The data of training cohort was used to establish the nomogram. The endpoint was OS, which was measured from the date of first diagnosis to the date of any cause of death. Survival was estimated using the Kaplan-Meier method and Cox regression analysis. The factors observed to have significant associations with OS were applied to construct the nomogram of OS.

The nomogram was internally and externally validated with 1,000 bootstrap resamples. Calibration curves were created using the marginal estimate and the model average prediction probability. ROC methodology can be explicitly used to evaluate predictive performance (8).

### Statistical Analysis

The statistical analysis was performed using SPSS statistics 21 and R version 3.6.3. The bilateral *p* < 0.05 was regarded as significant.

## Results

### Clinical Characteristics of the Patients

In general, a total of 7,121 adult GI DLBCL patients were identified from the SEER database. Patients were randomized into two sequential cohorts: training (*n* = 5,697) set and validation (*n* = 1,424) set. Patient characteristics are shown in Table 1.

**Table 1:** Patient characteristics.

Characteristic	**Training cohort (*n* = 5,697)**	**Validation cohort (*n* = 1,424)**
**Age at diagnosis, years**		
Median ± SD	69.0 ± 15.5	70.5 ± 15.2
Range	18–104	18–105
**Sex**		
Male	3,490 (61.3%)	825 (57.9%)
Female	2,207 (38.7%)	599 (32.1%)
**Race**		
White	5,009 (87.9%)	1,258 (88.3%)
Black	418 (7.4%)	104 (7.3%)
East Asian	270 (4.7%)	62 (4.4%)
**Marriage**		
Single	2,355 (41.3%)	623 (43.8%)
Married	3,342 (58.7%)	801 (56.2%)
**Stage**		
I	2,491 (43.7%)	633 (44.5%)
II	1,535 (26.9%)	354 (24.9%)
III-IV	1,671 (29.4%)	437 (30.6%)
**Primary site**		
Stomach	3,017 (53.0%)	744 (52.2%)
Intestine	2,680 (47%)	680 (47.8%)
**Surgery**		
Yes	2,214 (38.9%)	557 (39.1%)
No	3,483 (61.1%)	867 (60.9%)

### OS and Significant Prognostic Factors in the Training Cohort

**Figure 1:**
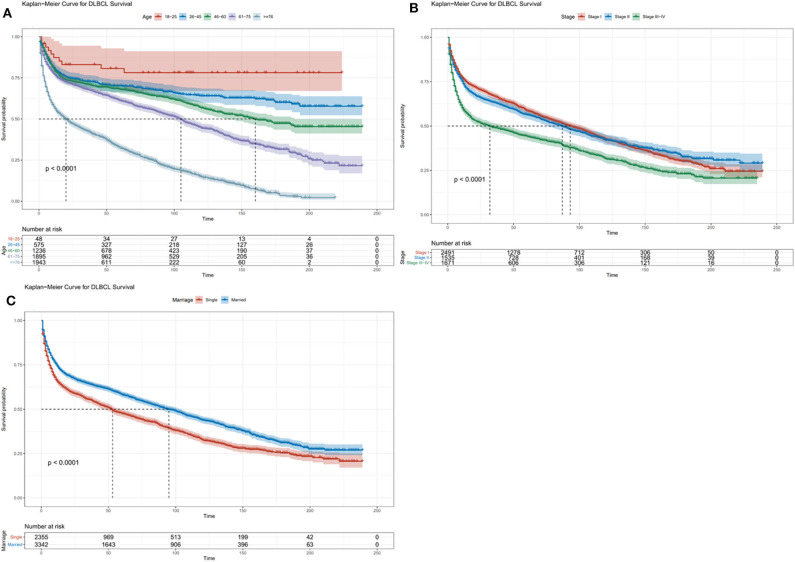
Kaplan-Meier survival curves for overall survival in the training cohort, as stratified by **(A)** Age, **(B)** Stage, **(C)** Marriage.

The median OS in the training cohort was 76 months (1–239 months), and 3, 5, and 10-year OS rates were 60.3, 53.9, and 39.5%. As shown in Figure 1, age at diagnosis, Ann Arbor stage, and marital status were important clinical predictors of OS. The results of the univariate and multivariate analysis are listed in Table 2.

**Table 2:** Survival analysis of the Training cohort.

**Variable**	**Univariate analysis**			**Multivariate analysis**		
	***P***	**HR**	**95%CI**	***P***	**HR**	**95%CI**
Age	<0.001	1.722	1.661–1.786	<0.001	1.739	1.677–1.803
Ann Arbor stage	<0.001	1.206	1.162–1.250	<0.001	1.266	1.221–1.313
Marital status	<0.001	0.741	0.697–0.788	<0.001	0.743	0.698–0.790

*HR, hazard ratio*.

### Prognostic Nomogram for OS

**Figure 2:**
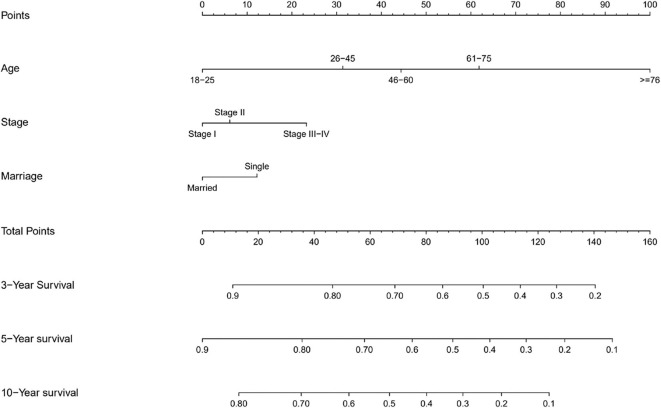
Nomograms for predicting the 3, 5, and 10-year OS.

**Figure 3:**
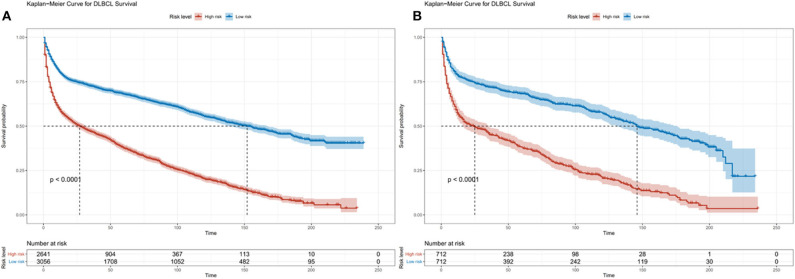
Kaplan-Meier survival curves for risk stratification in the training cohort **(A)** and validation cohort **(B)**.

The prognostic nomogram for 3, 5, and 10-year OS is shown in Figure 2. The OS was better for younger patients, patients with stage I disease, and married patients. With the help of the nomogram, patients were divided into different risk stratification to evaluate the OS (Figure 3).

### Validation of Predictive Accuracy of the Nomogram for OS

**Figure 4:**
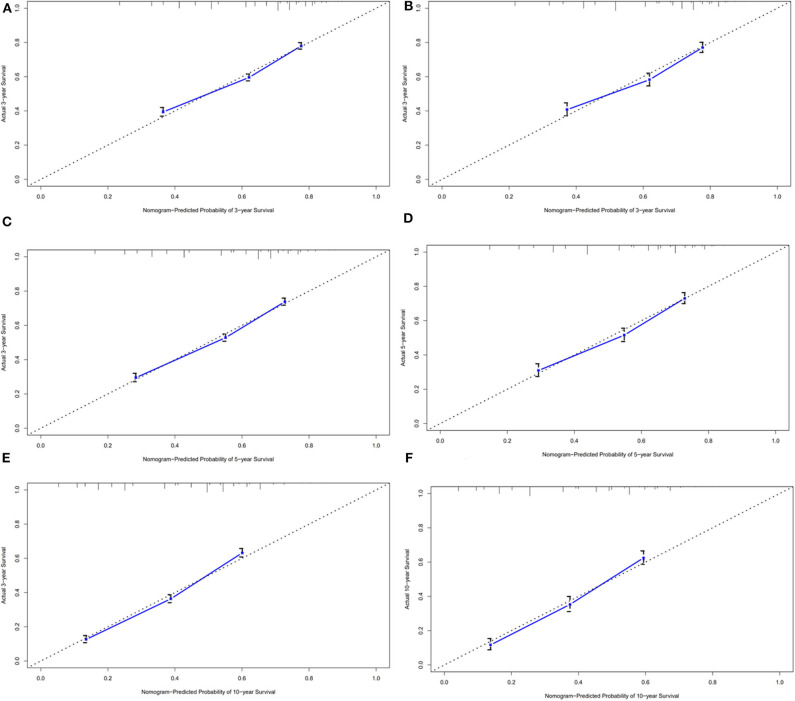
ROC curves for predictions of overall survival in the training cohort **(A,C,E)** and validation cohort **(B,D,F)** at 3, 5, and 10-year.

**Figure 5:**
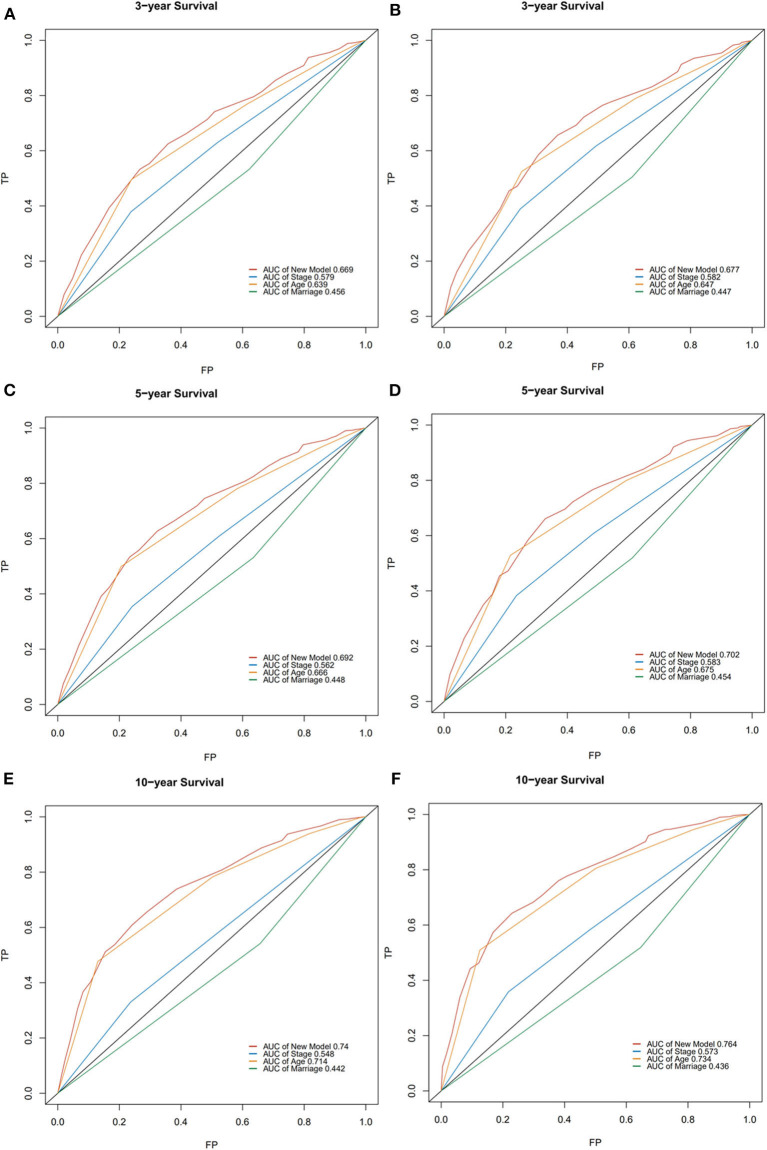
The calibration curves for predictions of overall survival in the training cohort **(A,C,E)** and validation cohort **(B,D,F)** at 3, 5, and 10-year.

In the validation cohort, the median OS was 74 months (1–236 months), and 3, 5, and 10-year OS rates were 59.7, 53.1, and 38.3%. The AUC of the nomogram for predicting 3, 5, and 10-year OS were 0.669, 0.692, and 0.740 (Figure 4). The internal and external calibration curves showed good optimal agreement between prediction by nomogram and observation in the probability of 3, 5, and 10-year survival (Figure 5).

## Discussion

Although primary GI DLBCL has been studied extensively in the past (9–12), its clinicopathological features are poorly described. Most DLBCL occur in patients above the age of 60, with a slight male predominance (1). We also observed this demographic feature in the present study. More and more studies showed that primary GI DLBCL had different clinical characteristics and treatment outcomes from nodal DLBCL (13–15). Some new prognostic evaluation systems are needed to identify high-risk patients. As a mathematical model based on graphic expression, the nomogram helps to determine the possibility of clinical event by combing clinical, pathological, and biological variables. The effects of several separate variables are integrated by the nomogram to give an individualized risk estimation for each patient. Compared to the traditional prognostic system, the nomogram showed better prediction in cancer population based on the SEER database (16–18). Nomograms are increasingly used for estimating lymphoma prognosis (19, 20).

To our knowledge, this is the largest retrospective case series of primary GI DLBCL with the aim to get a prognostic model to predict OS. In the present study, we developed a nomogram to predict the prognosis of patients with GI DLBCL, based on three significant factors: age at diagnosis, Ann Arbor stage, and marital status. Age is a well-known prognostic parameter for various cancers. As any other prognostic system (21–24), age at diagnosis was used as an independent prognostic factor. Refer to the age classification of NCCN-IPI (23), we divided patients into five age groups based on the data from the SEER database. Survival rates significantly decreased with increasing age at diagnosis.

Several staging systems have been developed over the past decades to improve prognostic stratification of NHL. The Ann Arbor staging system is widely used for staging of NHL. The Lugano staging system is a modification of the original Ann Arbor staging system designed for the staging of GI lymphoma. It was developed to incorporate measures of depth of invasion and distant nodal involvement. Ann Arbor classification is considered inadequate for the staging of GI DLBCL and the most widely used classification is the Lugano staging system which was adopted by the eighth edition of the Cancer Staging Manual of the American Joint Committee on Cancer (5). SEER database only provided data of Ann Arbor staging and our survival analysis showed that Ann Arbor staging was an independent prognostic index for GI DLBCL. Refer to Lugano classification, we only combined stage III and stage IV patients as one group, which is different from nodal DLBCL.

Marital status is not only a risk factor of developing cancers (25, 26), but also an independent prognostic indicator of many cancer (27–30). Married patients may possess relatively strong financial resources, which made it easier to get better therapies and thus was associated with better prognosis. Besides, they may also get additional care from their spouses. Le Guyader-Peyrou et al. found that marital status was independently associated with the 5-year relative survival of patients with DLBCL (31). However, socio-economic status was not associated with outcome (31). Our findings indicate that the prognosis of married patients with GI DLBCL is better than that of others. We did not have the information regarding socio-economical status that could be used for prognostication.

There are very few data illustrating the impact of IPI on primary GI DLBCL. The research results clarifying the prognostic effect of IPI on primary GI lymphoma were inconsistent. A retrospective multicenter clinical study of 299 B-cell lymphoma cases revealed IPI ≥2 to be an independent prognostic factor for worse OS (32). However, Shi et al. study of 137 patients found that there was no apparent prognostic significant correlation between IPI and survival (33). Lugano staging system was also used to modify the IPI for primary GI NHL (34, 35). Patients with a stage-modified IPI ≥2 had a median survival time (MST) of 44 months (34) and our study showed an MST of 28 months for higher risk patients. However, because of lack of data in SEER database, we cannot compare the proposed nomogram with IPI directly.

SEER database has many advantages with their strength primarily resting on larger sample size, inclusion of more diverse subsets of patients, and completed survival data. Our results must be interpreted carefully as there are some important limitations. First, Due to the nature of the SEER database, many clinical, pathological, and biological information regarding individual risk factors, such as IPI and some molecular markers were not available in the SEER database. Survival analysis was limited to a few factors and could not be further refined in high-risk patients. This issue is an important one to consider in other very large clinical data sets with direct ascertainment of these factors. The second limitation of this study is its retrospective nature, data integrity, and homogeneity are not guaranteed. Nevertheless, the patient population is relatively sufficient, and the findings of prognostic factors are consistent with other studies (31, 36, 37). Finally, treatment regimens of included patients were unclear. We only chose the data after 1996 so that most of patients might receive rituximab therapy. The use of new targeted agents might modify the clinical outcome of GI DLBCL (38, 39). Observational analyses using the SEER database can provide important hypothesis-generating data, from which future practice-changing prospective trials can be built.

In conclusion, the nomogram using age, Ann Arbor stage, and marital status permitted predictions about overall survival in adult patients with GI DLBCL. This predictive tool could help clinicians identify high-risk patients to improve their prognosis.

## Data Availability Statement

Publicly available datasets were analyzed in this study. This data can be found here: Surveillance, Epidemiology, and End Results (SEER) database (https://seer.cancer.gov/).

## Ethics Statement

Ethical review and approval was not required for the study on human participants in accordance with the local legislation and institutional requirements. Written informed consent for participation was not required for this study in accordance with the national legislation and the institutional requirements.

## Author Contributions

JW and MZ: had full access to all of the data in the study, took responsibility for the integrity of the data, the accuracy of the data analysis, statistical analysis, and drafting of the manuscript. RZ: analysis and interpretation of data. JX and BC: supervision. All authors: concept and design.

## Conflict of Interest

The authors declare that the research was conducted in the absence of any commercial or financial relationships that could be construed as a potential conflict of interest.

## References

[1] Foukas PG de Leval L. Recent advances in intestinal lymphomas. Histopathology. (2015) 66:112–36. 10.1111/his.12596 25639480

[2] Peng JC Zhong L Ran ZH. Primary lymphomas in the gastrointestinal tract. J Dig Dis. (2015) 16:169–76. 10.1111/1751-2980.12234 25678011

[3] Howell JM Auer-Grzesiak I Zhang J Andrews CN Stewart D Urbanski SJ. Increasing incidence rates, distribution and histological characteristics of primary gastrointestinal non-Hodgkin lymphoma in a North American population. Can J Gastroenterol. (2012). 26:452–6. 10.1155/2012/480160 22803021 PMC3395447

[4] Nakamura S Matsumoto T Iida M Yao T Tsuneyoshi M. Primary gastrointestinal lymphoma in Japan: a clinicopathologic analysis of 455 patients with special reference to its time trends. Cancer. (2003) 97:2462–73. 10.1002/cncr.11415 12733145

[5] Olszewska-Szopa M Wróbel T. Gastrointestinal non-Hodgkin lymphomas. Adv Clin Exp Med. (2019) 28:1119–24. 10.17219/acem/94068 31414733

[6] Choi SY Kim SJ Kim WS Kim K Ko YH. Aggressive B cell lymphomas of the gastrointestinal tract: clinicopathologic and genetic analysis. Virchows Arch. (2011) 459:495–502. 10.1007/s00428-011-1153-3 22002677

[7] López-Guillermo A Colomo L Jiménez M Bosch F Villamor N Arenillas L. Diffuse large B-cell lymphoma: clinical and biological characterization and outcome according to the nodal or extranodal primary origin. J Clin Oncol. (2005) 23:2797–804. 10.1200/JCO.2005.07.155 15728226

[8] Chambless LE Diao G. Estimation of time-dependent area under the ROC curve for long-term risk prediction. Stat Med. (2006) 25:3474–86. 10.1002/sim.2299 16220486

[9] Ishikawa E Nakamura M Shimada K Tanaka T Satou A Kohno K. Prognostic impact of PD-L1 expression in primary gastric and intestinal diffuse large B-cell lymphoma. J Gastroenterol. (2020) 55:39–50. 10.1007/s00535-019-01616-3 31493237

[10] Hallas C Preukschas M Tiemann M. Immunohistochemical distinction of ABC and GCB in extranodal DLBCL is not reflected in mutation patterns. Leuk Res. (2019) 76:107–11. 10.1016/j.leukres.2018.10.003 30360939

[11] Hori Y Yamamoto H Nozaki Y Torisu T Fujiwara M Taguchi K. Colorectal diffuse large B-cell lymphoma: molecular subclassification and prognostic significance of immunoglobulin gene translocation. Hum Pathol. (2020) 96:67–78. 10.1016/j.humpath.2019.09.003 31734190

[12] Bai ZM Li Z Guan T Wang LY Wang JR Wu SH. Primary gastric diffuse large B-cell lymphoma: prognostic factors in the immune-oncology therapeutics era. Turk J Haematol. (2020). 10.4274/tjh.galenos.2020.2019.0332. [Epub ahead of print]. 32160735 PMC7463217

[13] Kim SJ Kang HJ Kim JS Oh SY Choi CW Lee SI. Comparison of treatment strategies for patients with intestinal diffuse large B-cell lymphoma: surgical resection followed by chemotherapy versus chemotherapy alone. Blood. (2011) 117:1958–65. 10.1182/blood-2010-06-288480 21148334

[14] Wu YX Liu B Chen L Li JH Chen SQ. Prognostic factors of primary gastric diffuse large B cell lymphoma: a retrospective study of 75 cases in China. Ann Hematol. (2013) 92:861–2. 10.1007/s00277-012-1646-4 23238898

[15] Shi Y Han Y Yang J Liu P He X Zhang C. Clinical features and outcomes of diffuse large B-cell lymphoma based on nodal or extranodal primary sites of origin: analysis of 1,085 WHO classified cases in a single institution in China. Chin J Cancer Res. (2019) 31:152–61. 10.21147/j.issn.1000-9604.2019.01.10 30996573 PMC6433587

[16] Zhou H Shen J Zhang Y Huang Y Fang W Yang Y. Risk of second primary malignancy after non-small cell lung cancer: a competing risk nomogram based on the SEER database. Ann Transl Med. (2019) 7:439. 10.21037/atm.2019.09.01 31700875 PMC6803229

[17] Xiong Y Cao H Zhang Y Pan Z Dong S Wang G. Nomogram-predicted survival of breast cancer brain metastasis: a SEER-based population study. World Neurosurg. (2019) 128:e823–34. 10.1016/j.wneu.2019.04.262 31096027

[18] Zhang H Ma G Du S Sun J Zhang Q Yuan B. Nomogram for predicting cancer specific survival in inflammatory breast carcinoma: a SEER population-based study. PeerJ. (2019) 7:e7659. 10.7717/peerj.7659 31576238 PMC6752187

[19] Yang Y Zhang YJ Zhu Y Cao JZ Yuan ZY Xu LM. Prognostic nomogram for overall survival in previously untreated patients with extranodal NK/T-cell lymphoma, nasal-type: a multicenter study. Leukemia. (2015) 29:1571. 10.1038/leu.2015.44 25697894

[20] Zhong H Chen J Cheng S Chen S Shen R Shi Q. Prognostic nomogram incorporating inflammatory cytokines for overall survival in patients with aggressive non-Hodgkin's lymphoma. EBioMedicine. (2019) 41:167–74. 10.1016/j.ebiom.2019.02.048 30827933 PMC6443577

[21] International Non-Hodgkin's Lymphoma Prognostic Factors Project. A predictive model for aggressive non-Hodgkin's lymphoma. N Engl J Med. (1993) 329:987–94. 10.1056/NEJM199309303291402 8141877

[22] Sehn LH Berry B Chhanabhai M Fitzgerald C Gill K Hoskins P. The revised International Prognostic Index (R-IPI) is a better predictor of outcome than the standard IPI for patients with diffuse large B-cell lymphoma treated with R-CHOP. Blood. (2007) 109:1857–61. 10.1182/blood-2006-08-038257 17105812

[23] Zhou Z Sehn LH Rademaker AW Gordon LI Lacasce AS Crosby-Thompson A. An enhanced International Prognostic Index (NCCN-IPI) for patients with diffuse large B-cell lymphoma treated in the rituximab era. Blood. (2014) 123:837–42. 10.1182/blood-2013-09-524108 24264230 PMC5527396

[24] Wang J Zhou M Xu JY Yang YG Zhang QG Zhou RF. MYC and BCL-2 adjusted-International Prognostic Index (A-IPI) is a better predictor of outcome than the standard IPI for patients with diffuse large B-cell lymphoma treated with R-CHOP. Histol Histopathol. (2016) 31:285–92. 10.14670/HH-11-673 26424560

[25] Li M Han M Chen Z Tang Y Ma J Zhang Z. Does marital status correlate with the female breast cancer risk? A systematic review and meta-analysis of observational studies. PLoS ONE. (2020) 15:e0229899. 10.1371/journal.pone.0229899 32134997 PMC7058335

[26] Trudel-Fitzgerald C Poole EM Sood AK Okereke OI Kawachi I Kubzansky LD. Social integration, marital status, and ovarian cancer risk: a 20-Year prospective cohort study. Psychosom Med. (2019) 81:833–40. 10.1097/PSY.0000000000000747 31592935 PMC6832885

[27] Lau SKM Gannavarapu BS Carter K Gao A Ahn C Meyer JJ. Impact of socioeconomic status on pretreatment weight loss and survival in non-small-cell lung cancer. J Oncol Pract. (2018) 14:e211–20. 10.1200/JOP.2017.025239 29558251 PMC5951295

[28] Osazuwa-Peters N Christopher KM Cass LM Massa ST Hussaini AS Behera A. What's Love Got to do with it? Marital status and survival of head and neck cancer. Eur J Cancer Care. (2019) 28:e13022. 10.1111/ecc.13022 30784126

[29] Celeng C Takx RAP Lessmann N Maurovich-Horvat P Leiner T Išgum I The association between marital status, coronary computed tomography imaging biomarkers, and mortality in a lung cancer screening population. J Thorac Imaging. (2019) 35:204–9. 10.1097/RTI.0000000000000457 31651690

[30] Yang CC Cheng LC Lin YW Wang SC Ke TM Huang CI. The impact of marital status on survival in patients with surgically treated colon cancer. Medicine. (2019) 98:e14856. 10.1097/MD.0000000000014856 30882684 PMC6426559

[31] Le Guyader-Peyrou S Orazio S Dejardin O Maynadié M Troussard X Monnereau A. Factors related to the relative survival of patients with diffuse large B-cell lymphoma in a population-based study in France: does socio-economic status have a role? Haematologica. (2017) 102:584–92. 10.3324/haematol.2016.152918 27909221 PMC5394966

[32] Chen Y Chen Y Chen S Wu L Xu L Lian G. Primary gastrointestinal lymphoma: a retrospective multicenter clinical study of 415 cases in chinese province of guangdong and a systematic review containing 5075 Chinese patients. Medicine. (2015) 94:e2119. 10.1097/MD.0000000000002119 26632732 PMC5059001

[33] Shi Z Ding H Shen QW Lu XG Chen JY Chen X. The clinical manifestation, survival outcome and predictive prognostic factors of 137 patients with primary gastrointestinal lymphoma (PGIL): Strobe compliant. Medicine. (2018) 97:e9583. 10.1097/MD.0000000000009583 29505542 PMC5943112

[34] Huang J Jiang W Xu R Huang H Lv Y Xia Z. Primary gastric non-Hodgkin's lymphoma in Chinese patients: clinical characteristics and prognostic factors. BMC Cancer. (2010) 10:358. 10.1186/1471-2407-10-358 20604963 PMC2914701

[35] Li X Shen W Cao J Wang J Chen F Wang C. Treatment of gastrointestinal diffuse large B cell lymphoma in China: a 10-year retrospective study of 114 cases. Ann Hematol. (2012) 91:1721–9. 10.1007/s00277-012-1507-1 22733613

[36] Wästerlid T Harrysson S Andersson TM Ekberg S Enblad G Andersson PO. Outcome and determinants of failure to complete primary R-CHOP treatment for reasons other than non-response among patients with diffuse large B-cell lymphoma. Am J Hematol. (2020) 95:740–8. 10.1002/ajh.25789 32180274

[37] Lim RMH Chan NPX Khoo LP Cheng CL Tan L Poon EYL. A clinico-genotypic prognostic index for *de novo* composite diffuse large b-cell lymphoma arising from follicular lymphoma in Asian patients treated in the rituximab era. Sci Rep. (2020) 10:4373. 10.1038/s41598-020-61378-4 32152442 PMC7062756

[38] Wilson WH Young RM Schmitz R Yang Y Pittaluga S Wright G. Targeting B cell receptor signaling with ibrutinib in diffuse large B cell lymphoma. Nat Med. (2015) 21:922–6. 10.1038/nm.3884 26193343 PMC8372245

[39] Dunleavy K Erdmann T Lenz G. Targeting the B-cell receptor pathway in diffuse large B-cell lymphoma. Cancer Treat Rev. (2018) 65:41–6. 10.1016/j.ctrv.2018.01.002 29549872

